# Structure solution of DNA-binding proteins and complexes with *ARCIMBOLDO* libraries

**DOI:** 10.1107/S1399004714007603

**Published:** 2014-05-30

**Authors:** Kevin Pröpper, Kathrin Meindl, Massimo Sammito, Birger Dittrich, George M. Sheldrick, Ehmke Pohl, Isabel Usón

**Affiliations:** a University of Göttingen, Germany; b Instituto de Biologia Molecular de Barcelona (IBMB-CSIC), Spain; c Durham University, England; d Institucio Catalana de Recerca i Estudis Avancats (ICREA), Spain

**Keywords:** protein–DNA complexes and macromolecule structure solutions, structure-solution pipelines, molecular replacement, density modification

## Abstract

Protein–DNA interactions play a major role in all aspects of genetic activity within an organism, such as transcription, packaging, rearrangement, replication and repair. The molecular detail of protein–DNA interactions can be best visualized through crystallography, and structures emphasizing insight into the principles of binding and base-sequence recognition are essential to understanding the subtleties of the underlying mechanisms. An increasing number of high-quality DNA-binding protein structure determinations have been witnessed despite the fact that the crystallographic particularities of nucleic acids tend to pose specific challenges to methods primarily developed for proteins. Crystallographic structure solution of protein–DNA complexes therefore remains a challenging area that is in need of optimized experimental and computational methods. The potential of the structure-solution program *ARCIMBOLDO* for the solution of protein–DNA complexes has therefore been assessed. The method is based on the combination of locating small, very accurate fragments using the program *Phaser* and density modification with the program *SHELXE*. Whereas for typical proteins main-chain α-helices provide the ideal, almost ubiquitous, small fragments to start searches, in the case of DNA complexes the binding motifs and DNA double helix constitute suitable search fragments. The aim of this work is to provide an effective library of search fragments as well as to determine the optimal *ARCIMBOLDO* strategy for the solution of this class of structures.

## Abstract 2

The structure solution of DNA-binding protein structures and complexes based on the combination of location of DNA-binding protein motif fragments with density modification in a multi-solution frame is described.

## 1. Introduction   

DNA-binding proteins play essential roles in all aspects of transcription, DNA repair and gene regulation, and therefore it is no surprise that 6–7% of all proteins expressed in eukaryotic genomes have been estimated to interact with DNA (Luscombe *et al.*, 2000 ▶). Crystal structures of DNA-binding proteins alone and in complex with their target DNA sequences are an indispensible tool to decipher the diverse activation mechanisms as well as the structural basis of sequence-dependent DNA recognition (Stoddard, 2011 ▶; Tan & Davey, 2011 ▶; Lilley, 2010 ▶). A number of co-crystal structures showed early on that nature has evolved to use a limited set of structural domains for DNA recognition, and accordingly DNA-binding proteins have been classified into eight major groups based on their structure and function (Luscombe *et al.*, 2000 ▶). Although the number and diversity of DNA-binding structures solved in the last decade has greatly increased, most proteins still fall into one of these groups, which include the helix–turn–helix (HTH), zinc-coordinating, zipper-type, other α-helical and β-type proteins (Luscombe *et al.*, 2000 ▶).

Crystal structure determination of DNA-binding proteins generally follows the same protocols as for other soluble proteins. Protein–DNA complexes, on the other hand, often pose specific challenges. Crystallization is complicated by the fact that frequently many synthetic DNA oligonucleotides differing in length and/or sequence are tested. Crystals tend to be more fragile and radiation-sensitive owing to the increased absorption of heavier atoms. Diffraction patterns are often anisotropic owing to base stacking and the formation of semi-continuous DNA helices throughout the crystal, and the resolution is generally limited. The average resolution of 835 protein–DNA complexes classified as enzymes or regulatory proteins in the Nucleic Acid Database (Berman *et al.*, 1992 ▶) is approximately 2.5 Å, compared with approximately 2.2 Å for the entire Protein Data Bank (calculated using the *PDB-Metrics* server; Fileto *et al.*, 2006 ▶). More strikingly, there are only seven protein–DNA complexes determined at resolutions of 1.5 Å or better (0.8% compared with 6.1% for the entire PDB), and no crystal structures at the atomic resolution of 1.2 Å or better.

Current methods for solution of the phase problem often require the generation of crystals containing either brominated DNA oligonucleotides or selenomethionine-substituted proteins and hence additional experiments in the form of SAD and/or MAD methods (Hendrickson, 1991 ▶; Raghunathan *et al.*, 1997 ▶). Furthermore, only a few auto-tracing algorithms have so far been developed for nucleotides (Gruene & Sheldrick, 2011 ▶; Hattne & Lamzin, 2008 ▶; Pavelcik & Schneider, 2008 ▶; Cowtan, 2012 ▶). RNA secondary-structure elements have been used as multiple search fragments within an effective method combining manual map inspection, refinement, density modification and composite OMIT maps (Robertson & Scott, 2008 ▶; Robertson *et al.*, 2010 ▶). In order to enable structure solution from the native data set alone, we suggest taking advantage of the specific patterns of DNA-binding proteins to generate databases of conserved structural motifs and domains that can be used in a combination of fragment location with *Phaser* (McCoy *et al.*, 2007 ▶) and density modification and auto-tracing with *SHELXE* (Sheldrick, 2008 ▶, 2010 ▶), as implemented in *ARCIMBOLDO* (Rodríguez *et al.*, 2009 ▶).

We started with the structurally highly conserved domains that comprise the zinc-coordinating groups (also designated zinc-fingers) that are typically found in eukaryotic transcription factors, the helix–turn–helix group, which is found in many bacterial regulators (including the winged-helix motif; Huffman & Brennan, 2002 ▶), and zipper-type proteins. The family of β-type DNA-binding proteins was excluded as they show too much structural variability to be useful as fragments. TATA-box binding proteins, on the other hand, are structurally similar enough to be used in classical molecular-replacement approaches (Burley, 1996 ▶).

For proteins, main-chain α-helices provide the ideal, almost ubiquitous, small search fragment that will accurately match most helices present in the target protein with an r.m.s.d. below 0.5 Å. Most recently, general composite fragments, such as parallel–antiparallel arrangements of three strands or two helices, have been successfully used in *ab initio* phasing and implemented in our program. *BORGES* (Sammito *et al.*, 2013 ▶) extracts and clusters all possible fragments found in the PDB (Berman *et al.*, 2003 ▶) matching a given template to build a customized library. Starting from large collections of geo­metrical hypotheses (several thousands of clusters), the best-scoring ones at the fast fragment-location stages are further pursued through the slower iterative density modification and autotracing. In the case of protein–DNA complexes, the structurally conserved binding motifs and the DNA double helix constitute obvious potential search fragments. Although our method can address many difficulties in determining protein–DNA structures, the systematically lower resolution still remains a challenge. In this work, we present a study of the use of *ARCIMBOLDO* on the main types of DNA-binding proteins, an account of its optimal use and requirements for phasing within this scenario, and suggested parameterization derived from extensive testing on manually selected libraries. A pre-calculated library of suitable search fragments and data for a tutorial can be downloaded from http://chango.ibmb.csic.es/DNA.

## 2. Experimental   

For this study, we focused on the following prominent families of DNA-binding proteins: (I) zinc-coordinating, (II) helix–turn–helix (short HTH) and (III) zipper-type fragments. These domains can usually be identified based on their sequences even if they form part of a larger unknown protein. Initially, subsets of model fragments were extracted from PDB structures belonging to these DNA-binding protein families (I–III; for example, see Figs. 2, 4 and 7; Blundell *et al.*, 2006 ▶, Luscombe *et al.*, 2000 ▶). Models were further truncated to their constituent DNA-recognition domains to represent common characteristic protein–DNA interactions and for the generation of suitable fragments with sufficient accuracy yet that are large enough to render positive molecular-replacement and expansion results. Suitable zinc-finger, HTH and zipper-type target structures between 1.7 and 2.4 Å resolution were chosen from the Protein Data Bank (http://www.pdb.org; Berman *et al.*, 2003 ▶) as described in detail below.

### 2.1. Fragment database for structure solution   

Models for each of the three groups were obtained using the following protocol. Firstly, one representative structure determined at a minimum resolution of 2.4 Å with good crystallographic statistics and deposited structure factors was selected manually. The DNA-binding motif of this structure with a minimum length of 30 residues was then used to identify all similar structures in the Protein Data Bank using the *DALI* server (Holm & Rosenström, 2010 ▶), thus ensuring that no similar structure was missed owing to incomplete annotation. From this list, approximately 30 fragments with a root-mean-square deviation (r.m.s.d.) of no more than 2.0 Å from the starting fragment were inspected and manually selected using *Coot* (Emsley *et al.*, 2010 ▶) to avoid duplicates (for example, single-site variants of the same protein or the same protein bound in the same way to different target DNA oligonucleotides) and to ensure a diverse set of fragments for structure solution. On the other hand, various NCS-related copies of the same structure were left in the library sets as replicates in the case of the zinc-fingers 1f2i, 1llm, 1mey, 1un6, 2il3, 1hgh, 3mjh and 1g2d. The list of PDB files used to generate the database for each of the three cases is given in the Supporting Information^1^ (Supplementary Tables S1–S4).

The group of zinc-coordinating DNA-binding proteins was represented by Krueppel-like factor 4 (KLF4), which belongs to the SP/Klf family of eukaryotic zinc-finger transcription factors (Schuetz *et al.*, 2011 ▶). This structure was determined to a resolution of 1.7 Å.

The zipper-type representative chosen was the high-resolution crystal structure of C/EBP Bzip homodimer V285A variant bound to DNA, for which diffraction data to a resolution of 1.8 Å were available (PDB entry 2E42). It should be noted that there are currently only 27 zipper-type co-crystal structures in the Nucleic Acid Database.

The third group of HTH proteins represents a greater challenge for a number of reasons. The HTH motif is usually a small part of the entire protein and unlike several zinc-fingers has so far not been crystallized as one single domain bound to DNA. Therefore, the entire protein–DNA complexes are usually considerably larger and diffraction data rarely extend beyond 2.8 Å resolution. In order to assess the effect of resolution limitations, three target complexes were selected. We used the structure of the diphtheria toxin repressor (DtxR) without DNA determined at a resolution of 2.2 Å (Pohl *et al.*, 1998 ▶) as the starting point for database generation. DtxR has been solved in complex with DNA only to the medium resolution of 3.0 Å Bragg spacing, which is probably out of the range for this method (White *et al.*, 1998 ▶; Pohl *et al.*, 1999 ▶). However, the DtxR orthologue IdeR (iron-dependent regulator) from *Mycobacterium tuberculosis*, which shares a sequence identity of 57% (Schmitt *et al.*, 1995 ▶), has been solved at a resolution of 2.4 Å (Wisedchaisri *et al.*, 2007 ▶) and is used as a test case as described below (PDB entry 2ISZ). The DNA-binding domain of DnaA from *M. tuberculosis* in complex with box 1 DNA (PDB entry 3PVV), for which data in space group *P*3_2_21 to a resolution of 2.0 Å have been deposited (Tsodikov & Biswas, 2011 ▶), and the human homeobox protein Nkx-2.5 (PDB entry 3RKQ) crystallized in space group *P*6_5_, with data available to a resolution of 1.7 Å (Pradhan *et al.*, 2012 ▶), were also used as test cases.

### 2.2. *ARCIMBOLDO* workflow   

**Figure 1:**
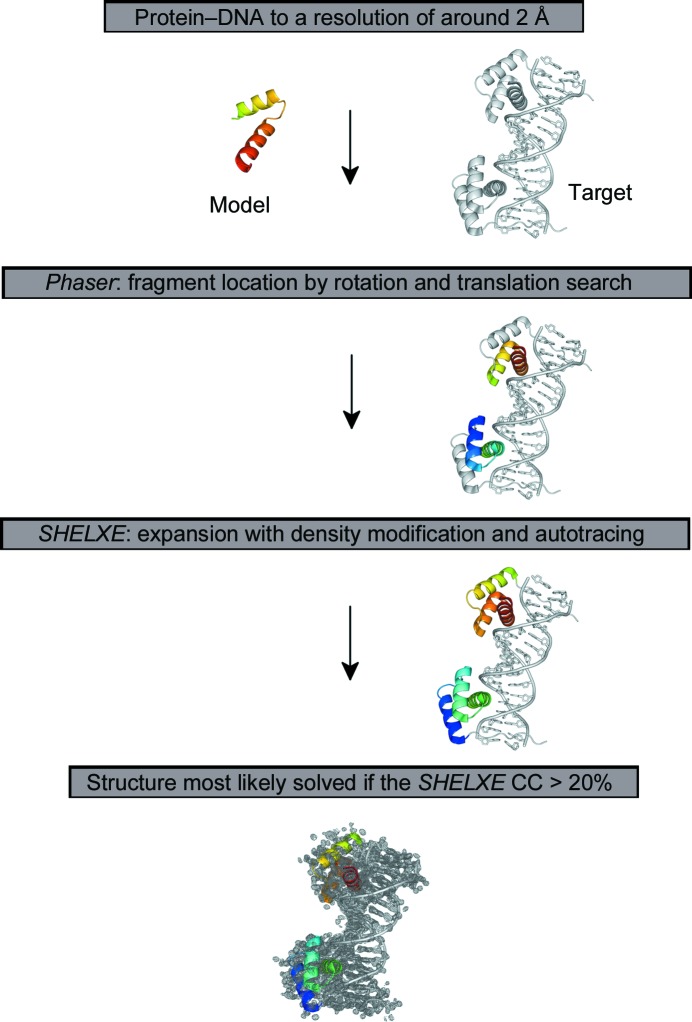
*ARCIMBOLDO* operated workflow starting from fragment subsets as initial molecular-replacement models assigned to *Phaser*, which performs a rotation and translation search including a subsequent refinement. Depending on the *ARCIMBOLDO* setup, all molecular-replacement results or results better than a specified average will be passed automatically to *SHELXE*. After subsequent and iterative density modification and auto-tracing, successful *SHELXE* expansion results could be identified by sorting the *SHELXE* CC (correlation coefficient) values. In our case of protein–DNA targets, CC values above 20% tagged a successful solution for a specific PDB start fragment.

The general workflow for *ARCIMBOLDO* (Rodríguez *et al.*, 2009 ▶, 2012 ▶) is shown in Fig. 1 ▶. The program was run for each of the fragments in the library, combining fragment location with *Phaser* v.2.1.4 (McCoy *et al.*, 2007 ▶) and density modification and auto-tracing of the top solutions with *SHELXE* v.2012 (Sheldrick, 2008 ▶, 2010 ▶) in order to expand the small substructures to a substantial and easily recognizable part of the polypeptide component of the structure. The runs were set up by searching for one or more copies of the fragments and by cutting the resolution for the fragment rotation search at 2–2.5 Å (depending on the data resolution of the targets). The molecular-replacement search was carried out stepwise with 1.5° rotation steps for the orientation search and 0.7 Å translation steps for the positional search. Packing filters and rigid-body refinement were also performed with *Phaser*. After each fragment-location step, expansion with no resolution cutoff is attempted on the ten solutions with the highest *Phaser* TFZ score characterizing their translation function. The parameters generally chosen for the *SHELXE* expansion are 30 cycles of density modification alternating with ten or 20 rounds of auto-tracing, no sharpening, deriving phases from the fragments to the resolution limit of 1.9 Å and extrapolating missing reflections up to 1.0 Å resolution using the free-lunch algorithm (Caliandro *et al.*, 2005 ▶; Yao *et al.*, 2006 ▶; Usón *et al.*, 2007 ▶). Deviations from the use of these parameters for the *SHELXE* expansion are described in detail in the corresponding sections. As in other phasing scenarios, a bimodal distribution in the correlation coefficient (CC; Fujinaga & Read, 1987 ▶) between the native intensities and those calculated from the main-chain trace rendered by *SHELXE* is a good indication that the structure has been solved. In the present work, solutions were verified by inspection of the electron-density map and calculation of the mean phase error (MPE) between the phases and those derived from the deposited models. Correct solutions correspond to CC values above 20%, as the main-chain trace is limited to the polypeptide fraction of the structure. *ARCIMBOLDO* is used running on a Condor grid with 240 cores on the FCSCL (http://www.fcsc.es) supercomputer CALENDULA, where the subset fragment jobs can be calculated in parallel (Tannenbaum *et al.*, 2002 ▶). A typical library run with the described parameters took 36 h, but setting it to stop after a solution has been achieved reduces the run time to a couple of hours.

## 3. Results and discussion   

### 3.1. Zinc-coordinating proteins   

**Figure 2:**
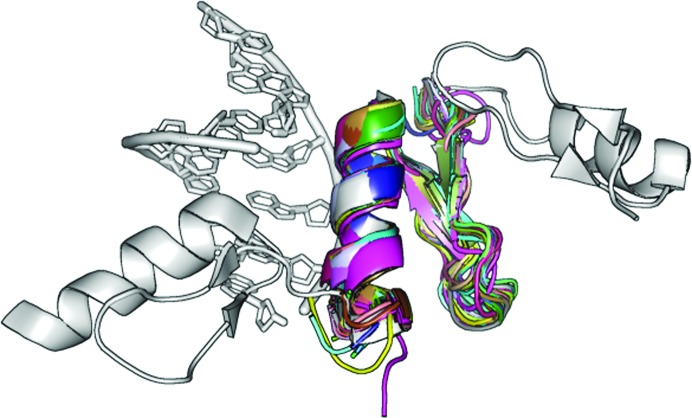
Zinc-coordinating protein target (grey) and zinc-finger fragments (rainbow). A zinc-finger DNA-binding protein at 1.7 Å resolution with PDB code 2WBS (space group *P*2_1_2_1_2_1_) was chosen from the PDB and used as a target structure (shown in grey). Zinc-finger fragment subsets aligned with the target are shown in rainbow.

Proteins containing zinc-coordination binding motifs constitute the largest single group of transcription factors in eukaryotic genomes. They typically present a structurally conserved characteristic zinc environment (Fig. 2 ▶) in which one or two Zn atoms are coordinated by cysteine and histidine residues in a tetrahedral geometry (Luscombe *et al.*, 2000 ▶). We can benefit from this common geometry of a small part of our target structure, as it can be predicted from the sequence.

The selected target is the zinc-finger structure with PDB code 2WBS determined in space group *P*2_1_2_1_2_1_, containing a seven base-pair double-stranded DNA helix surrounded by three connected zinc-finger fragments totalling 87 amino acids (Schuetz *et al.*, 2011 ▶). Diffraction data with a completeness of 99.4% to a resolution of 1.70 Å are available in this case.

#### 3.1.1. Zinc-coordinating motifs and *ARCIMBOLDO* results   

**Figure 3:**
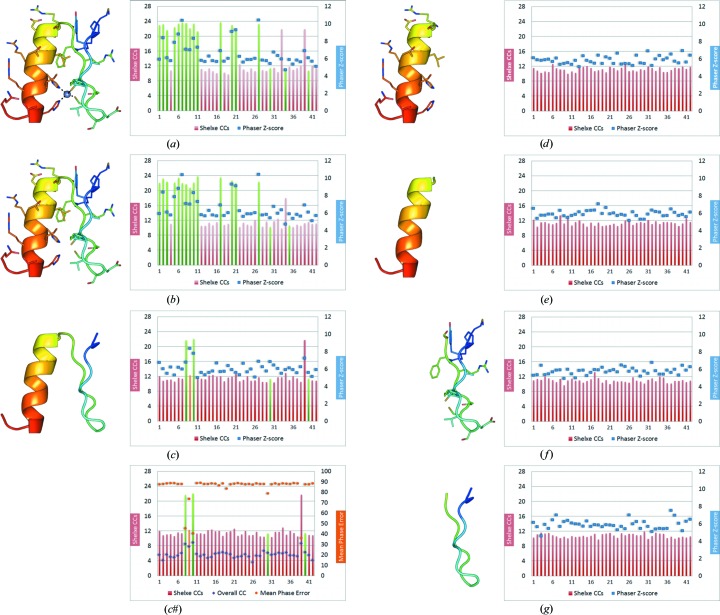
Zinc-finger fragments used as search models (PDB code 1f2i is shown as an example). The zinc-finger fragments were truncated stepwise during the target structure-solution procedure to investigate systematically the tradeoff between fragment completeness and accuracy of the binding motif for the solution of this class of proteins. The models used are shown in cartoon representation on the left and the *Phaser* and *SHELXE* results are shown in diagrams on the right, where the green and red bars represent the *SHELXE* CC and the blue squares represent the *Phaser* TFZ score (the PDB codes corresponding to the numbers on the *x* axis can be found in Table S1 of the Supporting Information): (*a*) zinc-finger fragment without truncation (27–31 amino acids; 30–35% of the original zinc-finger fragment), (*b*) fragment omitting the Zn atom, (*c*) side chain truncated to polyalanine residues spanning the whole zinc-finger motif, (*d*–*g*) fragment subsets containing only helix or β-strands with and without side chains: (*d*, *e*), 8–13 amino acids, 9–15% of the original zinc-finger fragment, (*f*, *g*) 13–16 amino acids, 15–18% of the original zinc-finger fragment. H atoms were always omitted from the different fragment subsets. Diagrams show *ARCIMBOLDO* runs started with a subset of zinc-finger fragments. Attempts in which *ARCIMBOLDO* succeeded in solving the PDB entry 2WBS target are shown as green *SHELXE* CC (correlation coefficient) values (fragment PDB codes are listed at the bottom). (*c*#) shows the OCC (overall correlation coefficient of the fragment before density modification) and final MPE (mean phase error) after density modification and auto-tracing with *SHELXE*. (*e*) shows fragment subsets truncated to polyalanine and only helix polyalanine cases. The use of helical or β-strand fragments themselves (for example, general fragments for *ab initio* structure solution with *ARCIMBOLDO*) does not lead to any feasible solutions. In contrast, retaining the motif but truncating the side chains (*c*) is successful in some cases. The smallest solving fragment represents 14.18% of the mass of the asymmetric unit.

Starting from 42 zinc-finger models, seven alternative fragment subsets sharing common structural patterns were derived (Fig. 2 ▶). As the efficiency of the method depends both on fragment size and deviation from the geometry in the target structure, the aim was to optimize the library of fragments. All sets were provided to *ARCIMBOLDO*, which starts by running *Phaser* in parallel using all search models. Normally, the initial results are scored and only selected models characterized by the best figures of merit (LLG/TFZ score of the first rotation and/or translation) are further pursued. In this study, each search model is fully tried in parallel for test purposes. For each fragment, solutions were sorted according to the TFZ score characterizing their translation function. Expansion through density modification and auto-tracing was attempted on the top ten solutions using our standard *SHELXE* parameters. In the case of zinc-coordinating motifs, stepwise truncation of the fragments was performed in order to systematically assess the need for conserved protein–DNA parts which lead to successful fragment location (Fig. 3 ▶). To achieve phasing starting from small fragments, a balance between correctness and completeness is critical: a minimum scattering power is needed for expansion to succeed but larger models tend to show increased an r.m.s.d. compared to the final structure, which hampers the process. With our approach, at 2 Å resolution successful expansion requires an accuracy of around 0.5 Å r.m.s.d. for a completeness of the main chain of around 10%.

As a first attempt, the whole motif (including the zinc ion and all side-chain atoms) was used for solving the target zinc-finger protein–DNA complex (PDB entry 2WBS). An overall 40% success rate (Fig. 3 ▶ *a*) was achieved. When omitting the zinc ion, phasing succeeds in one case fewer (Fig. 3 ▶ *b*). *Phaser* TFZ scores and *SHELXE* CC values for the final traced models correlate very well for high TFZ scores, invariably indicating solutions, but in most cases figures of merit at the fragment-search state cannot discriminate trials that will eventually develop into solutions. Conversely, low TFZ scores would often lead to the underestimation of a potentially useful start fragment for further *SHELXE* density modification and auto-tracing. As shown in Figs. 3 ▶(*a*) and 3 ▶(*b*), in the case of PDB fragments 1a1g and 1a1i (named after the PDB codes, where upper-case letters indicate the code for a test case and lower-case letters indicate the code for the source of a model) a TFZ score of about 6 turned into a solved structure after *SHELXE* with CC values above 22%, while for instance 2hgh with a TFZ score of 7 did not succeed. Further truncation to polyalanine search fragments reduced the success rate to approximately 10% (Figs. 3 ▶ *c* and 3*c*#)^2^. Although the success rate is reduced, up to this point all solutions exhibit a clear-cut discrimination between solved and unsolved. When further truncation is pursued to dismember the conserved zinc-finger motif into its helix and β-hairpin elements, no solution is achieved (see Figs. 3 ▶ *d*–3 ▶ *g*). Thus, the small motif succeeds where the isolated secondary-structure elements do not.

It should be noted that during *ARCIMBOLDO* runs fixed settings were used for *SHELXE*, as changing these values directly influences the CC values and therefore the success rate might vary. The presence of DNA in our target structure somewhat complicates autotracing in the standard *SHELXE* v.2012. On one hand the procedure creates and places a polyalanine model well at the appropriate zinc-finger protein position. On the other hand *SHELXE* also starts to trace β-strands across the phosphate backbone and additionally places short α-helices onto nucleotides. This behaviour decreases the accuracy of the model owing to the application of protein structural restraints to nucleobases, sugar and phosphate groups, which primarily leads to more inaccurate phases and therefore handicaps further iterative structure solution *via SHELXE*.

In summary, whereas the smaller, less specific secondary-structure models such as a single α-helix or strands are not sufficient to phase the structure, the complete zinc-finger motif constitutes a suitable search fragment. Even a main-chain-trimmed fragment is effective in solving our target structure.

### 3.2. Zipper-type proteins   

**Figure 4:**
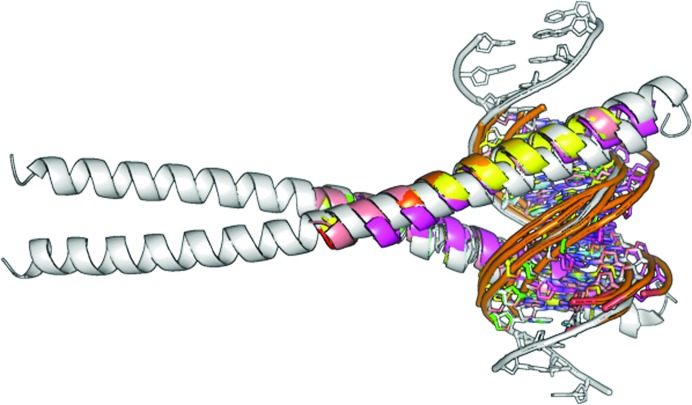
Zipper-type protein target (grey) and zipper-type fragments (rainbow). A zipper-type protein at 1.8 Å resolution with PDB code 2E42 was used as the target structure. Zipper-type fragment subsets aligned to the target are shown in rainbow.

Leucine zippers are parallel α-helical coiled-coil motifs and as such are one of the most common mediators of protein–protein interactions (Nair & Burley, 2006 ▶). They derive their name from their manner of dimerization, which is mediated through the formation of a coiled coil by a 30-amino-acid section at the end of each helix (Fig. 4 ▶). The zipper region consists of leucine or a similar hydrophobic amino acid at every seventh residue position in the α-helix. The most widely known leucine-zipper (LZ) proteins are the basic region leucine zippers (bZIPs; Luscombe *et al.*, 2000 ▶; Nikolaev *et al.*, 2010 ▶). Just like the zinc-coordinating binding motifs, zipper-type motifs provide a characteristic search fragment.

#### 3.2.1. Zipper-type binding motifs and *ARCIMBOLDO* results   

**Figure 5:**
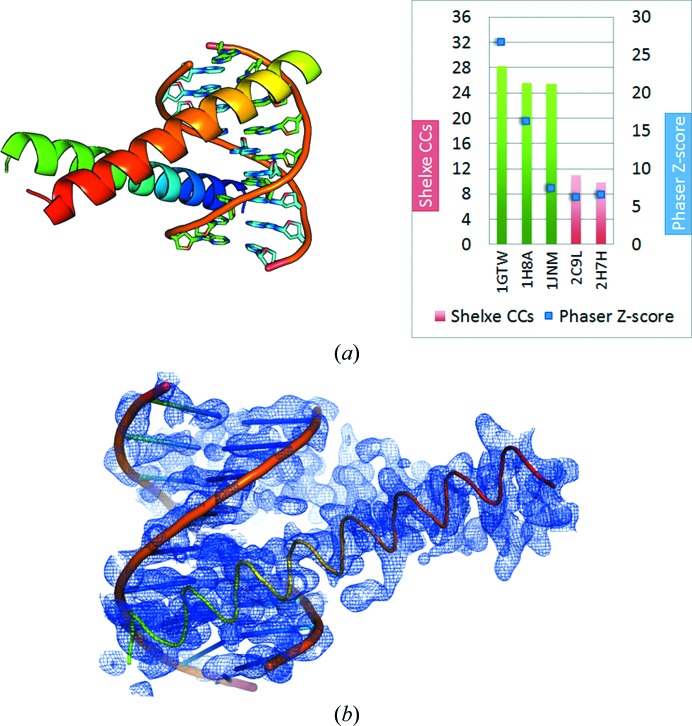
1GTW as a representative of the used zipper-type protein fragments for structure solution *via ARCIMBOLDO* (left). PDB codes 1gtw, 1h8a and 1jnm used as fragment subsets for zipper-type protein led to a solution after expansion (right, green bars) indicated by high *SHELXE* CC and *Phaser* TFZ scores for the solution. The *SHELXE* settings are -m30 -v0 -y1.9 -a10 -t30 -e1.0 -q -s0.67. (*b*) Detail of the resulting electron-density map after expansion of the best solution PDB starting fragment 1gtw is shown in blue at a 1σ contour level. The extrapolated data (free-lunch algorithm to 1.0 Å) were used in the displayed map. For illustration purposes a cartoon representation of the final model of the zipper-type protein complex (rainbow) was placed into the electron-density map, showing part of the asymmetric unit and highlighting the map quality.

The C/EPBβ homodimer (PDB entry 2E42) zipper-type protein–DNA complex determined at a resolution of 1.8 Å in space group *C*222_1_ was used as a target structure (Fig. 4 ▶, shown in grey). The asymmetric unit contains 16 base pairs and 130 amino acids. Zipper-type fragments from five model structures (1GTW, 1H8A, 1JNM, 2C9L and 2H7H) were used in the structure-solution pipeline without any further truncation of, for example, side chains. For zipper targets, part of the DNA was also taken into account (Fig. 5 ▶ *a*, left). After expansion with *SHELXE* (Fig. 5 ▶ *a*, right) three of the five fragments used (*i.e.* 1gtw, 1h8a and 1jnm) led to a successful solution (green) with high *SHELXE* CC values of up to 28% and TFZ scores above 25. These three models contain both the DNA and protein sequences that are most similar to the target structure. The resulting electron-density map (Fig. 5 ▶ *b*) after *SHELXE* expansion shows side chains, DNA sugars and phosphates as well as base-pair residues that are easily and unambiguously identified. Nevertheless, the *SHELXE* auto-tracing algorithm still tends to trace through the DNA, with the same consequences as discussed in §[Sec sec3.1.1]3.1.1. *SHELXE* is very accurate in placing and building polyalanine residues along the actual zipper α-helix positions.

**Figure 6:**
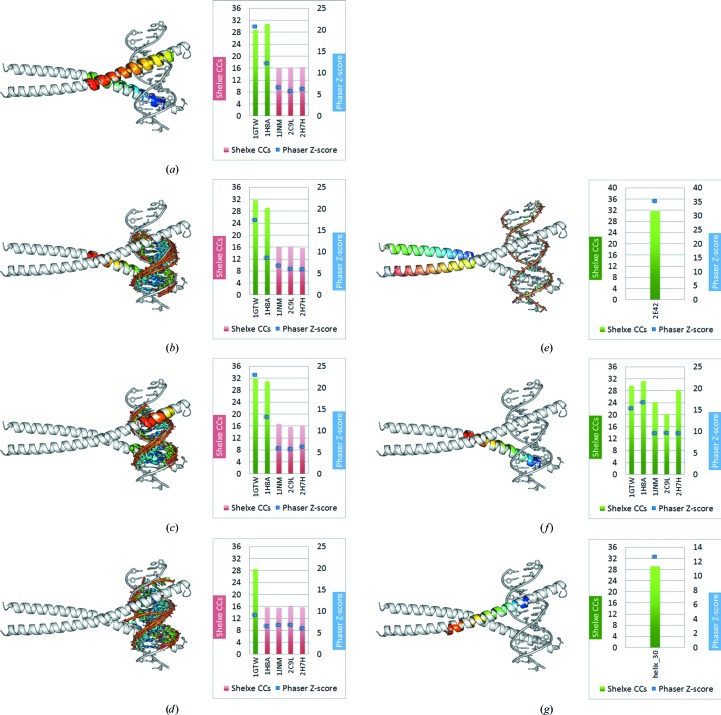
Zipper-type target 2E42 with modified models as input to *ARCIMBOLDO*: (*a*) using only the helices (both) from the models leads to solutions for just two (1gtw and 1h8a) of the five fragments; (*b*) using as search fragments just one long helix (30 amino acids) and the DNA fragment leads to solutions in only two of the five models (1gtw and 1h8a); (*c*) the same two fragments (1gtw and 1h8a) also lead to a solution if the DNA plus shorter helices (12 amino acids each) are used as search fragments; (*d*) using only the DNA of the models as a search fragment leads to a solution in only one case (1gtw); (*e*) using the DNA-distant helices taken from the target structure 2E42 as search fragments leads to a clear solution; (*f*) cutting down this fragment even more to just one helix without the DNA leads to a solution for all five of the models (1gtw, 1h8a, 1jnm, 2c9l and 2h7h); (*g*) even searching for two copies of a model helix of 30 amino acids leads to a solution as the DNA-binding part of the zipper helix is quite straight and does not deviate much from an ideal straight model helix. The smallest solving fragment represents 8.13% of the mass of the asymmetric unit.

In order to further investigate the conditions under which smaller models are suitable to phase the target structure, the starting models were stepwise trimmed to smaller fragments. Omitting the DNA leads to two successful solutions with 1gtw and 1h8a (Fig. 6 ▶ *a*). Truncating these two models to only one of the two α-helices with the DNA fragment (Fig. 6 ▶ *b*) or after reducing the length of the helices to 12 amino acids and keeping the DNA (Fig. 6 ▶ *c*) also results in successful structure solution, whereas all models derived from 1JNM, 2C9L and 2H7H failed. In the next step only the 7 bp double-stranded DNA was used as a search model to probe the suitability of DNA fragments alone. Phasing could only be achieved in the case of 1gtw, the sequence of which differs from the target structure in only one amino acid and two base pairs (Fig. 6 ▶ *d*). In order to further determine whether the DNA-binding region is crucial in solving the structure, the DNA-distant portion of the helix pairs (30 amino acids each as indicated in Fig. 6 ▶ *e*) was used as input to *ARCIMBOLDO*. This fragment clearly solves with a *Phaser* TFZ score of 35.21, a *SHELXE* CC of 31.71% and a final MPE of 41.70° (Fig. 6 ▶ *e*). Given the success with two helices, the search fragments were reduced to only one helix (30 amino acids long) and in this case phasing was achieved for all five model fragments (Fig. 6 ▶ *f*). In all five cases the target structure is clearly solved, but again the fragments based on 1gtw and 1h8a show the highest *Phaser* TFZ scores and *SHELXE* CC values (see Table 1 ▶). As the zipper-type DNA-binding helices are rather long (around 60 amino acids) even a single straight model helix of 30 amino acids is suitable to solve the structure when searching for two fragments, as the kink in the zipper helix is in the middle of the 60 amino acids and each of the two halves is straight and does not deviate much from an ideal helix (Fig. 6 ▶ *g*).

In summary, even if in favourable cases a single α-helix or even a DNA helix may already be sufficient to phase a leucine-zipper-type structure, a more complete binding motif fragment may be appropriate to solve larger cases provided that its geometry is close enough to the target.

### 3.3. Helix–turn–helix (HTH) proteins   

**Figure 7:**
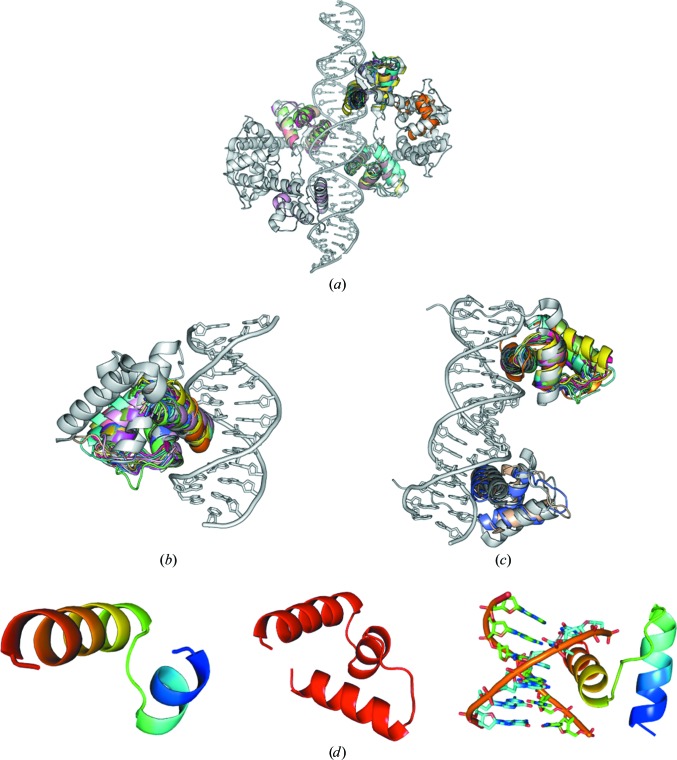
(*a*) Group of HTH-type protein test cases used and search models. Target 2ISZ (space group *P*1) consists of four HTH fragments coordinated to a rather long DNA double strand. HTH-type fragment subsets are aligned with the target (shown in rainbow). Helix–turn–helix proteins are shown in grey and HTH-type search fragments are shown in rainbow. (*b*) HTH-type protein at 2.0 Å resolution with one HTH-type binding motif (PDB entry 3PVV; space group *P*3_2_21) used as the target structure. All HTH-type fragment subsets are also aligned with the HTH target (rainbow). (*c*) HTH-type protein at 1.7 Å resolution with two HTH-type binding motifs (PDB entry 3RKQ; space group *P*6_5_) used as the target structure. (*d*) Left, HTH-type search fragments (rainbow); middle, three-helix bundle HTH starting fragment (red); right, DNA including HTH-type fragment subsets as a search fragment (rainbow).

Many transcription regulators as well as various enzymes from prokaryotes and eukaryotes take advantage of HTH motifs as a common DNA-recognition interface. The motif is characterized by a 20-amino-acid segment consisting of two almost perpendicular α-helices connected by a turn. The second helix, which is normally inserted into the major groove of B-DNA, is known as the recognition or probe helix, whereas the first α-helix stabilizes the interaction between protein and DNA but does not play a particularly strong role in its recognition (Matthews *et al.*, 1982 ▶). The helix–turn–helix motif is usually part of a three-helix bundle and in many cases is flanked by an additional small antiparallel β-sheet, also designated the winged-helix motif, which is present in the DtxR target structure (Ogata *et al.*, 1992 ▶; Huffman & Brennan, 2002 ▶). Supporting contacts with the DNA backbone are mostly made by the linker and the first α-helix (Fig. 7 ▶). Despite this predictable architecture, the HTH motifs tend to be more flexible, resulting in a less conserved starting model for the fragment search when compared with the more conserved and rigid zinc-finger or zipper-type motifs. In addition, the helices are rather short compared with the previous types.

#### 3.3.1. Helix–turn–helix (HTH) proteins and *ARCIMBOLDO* results   

The first target structure for an HTH protein (2ISZ) crystallized in space group *P*1 and data were available to a resolution of 2.4 Å (Wisedchaisri *et al.*, 2007 ▶). The structure is rather large as it contains 4 × 140 protein residues in the asymmetric unit binding to a 33 bp DNA (Fig. 7 ▶ *a*).

A second target structure with one HTH protein bound to a DNA fragment was used (3PVV) for which data in space group *P*3_2_21 to a resolution of 2.0 Å were available. The structure contains two monomers in the asymmetric unit, each composed of 96 amino acids and a 13 bp double-stranded DNA (Tsodikov & Biswas, 2011 ▶; Fig. 7 ▶ *b*). The third study case 3RKQ crystallized in space group *P*6_5_, where data were available to a resolution of 1.7 Å (Pradhan *et al.*, 2012 ▶). In this structure two HTH motifs are coordinated to a shorter DNA fragment compared with 2ISZ (115 protein residues and a 19 bp DNA in the asymmetric unit; Fig. 7 ▶ *c*). It is noteworthy that besides the HTH-motif proteins, large DNA helices are present in these structures and build up a major part compared with the protein HTH fragment itself.

The *ARCIMBOLDO* protocol was followed analogously to the cases of the zinc-coordination and zipper-type protein motifs. Subsets derived from an initial collection of 25 models were used as input fragments for *Phaser*. The parameters used for the *SHELXE* expansion as discussed in §§[Sec sec3.1.1]3.1.1 and [Sec sec3.2.1]3.2.1 are 30 cycles (up to 300 for special cases of density modification) alternating with ten or 20 rounds of auto-tracing. Sharpening was switched off. For 2ISZ the missing reflections were extrapolated using the free-lunch algorithm in *SHELXE* to 2.0 Å resolution. Solvent content also plays a critical role for *SHELXE* density modification and auto-tracing and was set at the value of the target structure PDB unit-cell contents. In our tests of HTH DNA-binding proteins, HTH, three-helix bundle HTH and also 6 bp DNA HTH motifs were used as fragment subsets (Fig. 7 ▶ *d*).

**Figure 8:**
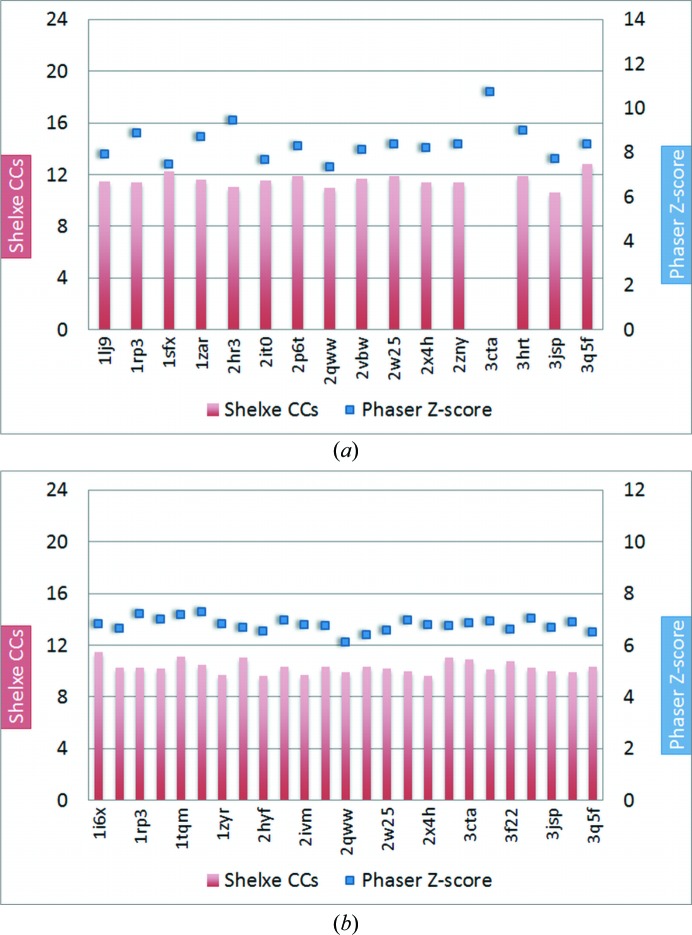
(*a*) Results for HTH-type protein 2ISZ as target after a four-fragment search (with HTH models) *via Phaser* at 2.4 Å and (*b*) HTH motif 3RKQ after search for two fragments. For both target structures no solution was found. The model with the missing entry for the CC bar in (*a*) (3cta) did not pass the packing in *Phaser*.

Although three different HTH targets of different complexity arising from their resolution and contents of the asymmetric unit were chosen for this investigation, none of them could be solved with our initial library by the *ARCIMBOLDO* routine, as shown in Fig. 8 ▶ for the cases with the best and the most limited resolutions and the subsets of largest fragments. In the case of the largest structure, with data to only 2.4 Å resolution, after a promising initial *Phaser* partial molecular-replacement fragment location with TFZ scores of up to 8, the structure could not be expanded by *SHELXE* from the starting phases provided by the partial structures, as can be seen from the low CC values of the final trace of around 12.

#### 3.3.2. HTH perfect models cut out from the target   

Since our first attempts did not succeed in phasing the target structure using the HTH motifs, we performed additional tests using original fragments directly cut out from the target structures in order to investigate the reason for the failure.

**Figure 9:**
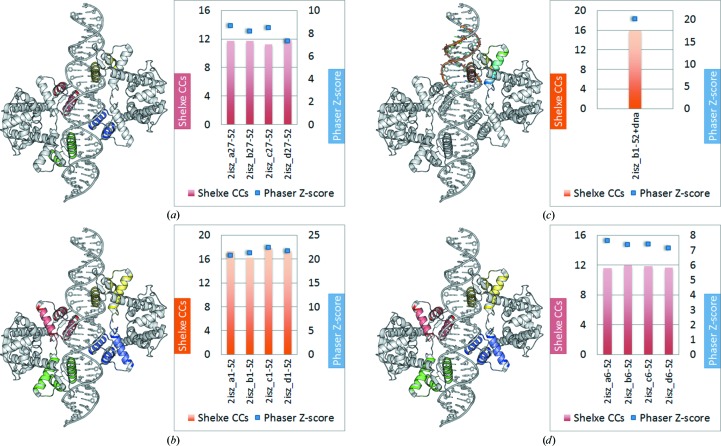
Phasing and expansion results from *ARCIMBOLDO* for HTH target 2ISZ with ideal search fragments. (*a*) HTH fragment (residues 27–52 from 2ISZ): *Phaser* TFZ scores in the range 7–9 and *SHELXE* CCs of 11–12%. (*b*) Three-helix bundle HTH fragment cut out from the target structure (residues 1–52 from 2ISZ): the *Phaser* TFZ scores are quite promising with values of around 20, but *SHELXE* correlation coefficients of <20% after density modification and auto-tracing indicate that *SHELXE* could not further improve the structure. (*c*) Three-helix bundle HTH fragment (52 residues) with a 10 bp DNA fragment: the *Phaser* TFZ scores are again around 20 but the *SHELXE* CCs are slightly lower (16%). (*d*) Trimmed three-helix bundle HTH fragment (highly flexible residues 1–5 removed) and all side chains set to alanine: the *Phaser* TFZ scores are drastically decreased to ∼8 and the *SHELXE* CCs remain <12%.

Firstly, tests with the helix–turn–helix fragment taken from the original target 2ISZ (residues 27–52) were performed. The *Phaser* TFZ scores after location of the fourth fragment again look rather promising (around 8); the initial mean phase error, however, is in the region of 90°. It is therefore not surprising that the final CC after density modification and auto-tracing with *SHELXE* (around 12%) and the final MPE (close to 90°) indicated that phasing had failed (Fig. 9 ▶ *a*).

For the three-helix bundle HTH fragment from 2ISZ (residues 1–52 from each of the four HTH chains) promising TFZ scores from *Phaser* (>20 after location of the fourth fragment) were obtained and the starting mean phase errors had values of around 60°, which shows that *Phaser* was able to correctly place the fragments; the final *SHELXE* correlation coefficients are slightly below 20% and the mean phase errors are stuck between 60 and 65° for the final trace (Fig. 9 ▶ *b*).

Increasing the search fragment to the three-helix bundle fragment from 2ISZ plus a small fragment of DNA (52 amino acids plus 10 bp DNA) leads to *Phaser* TFZ scores of higher than 20 after correct location of the second fragment and a starting MPE of around 60°, but the *SHELXE* CCs still remained at 16% after auto-tracing, with a final mean phase error of around 64° (Fig. 9 ▶ *c*). Again, *Phaser* succeeded in correctly locating the fragments but *SHELXE* could not expand to the rest of the structure from this starting point.

In a realistic scenario, the models can hardly be as close to the target structure as those taken directly from the final structure; in particular, the coordinates of side chains and flexible parts will deviate from prediction. To investigate how precise such small models are required to be under the size and resolution conditions of this case, the model was reduced to the main chain of residues 6–52. The first five highly flexible residues were omitted and all side chains were set to alanines. After location of the fourth fragment the *Phaser* TFZ scores are much lower than for the fragments with side chains (around 7–8) and the starting MPEs are close to 90°, *i.e. Phaser* did not correctly place the fragments. From this point, obviously *SHELXE* cannot trace the structure either and the final CCs remain at 11–12% (Fig. 9 ▶ *d*).

It is clear that the resolution of the target 2ISZ is too low for *SHELXE* to successfully expand the structure even from the ideal fragment. Furthermore, it is likely that the DNA part, which constitutes a large fraction of the total structure, is also interfering with protein tracing.

**Figure 10:**
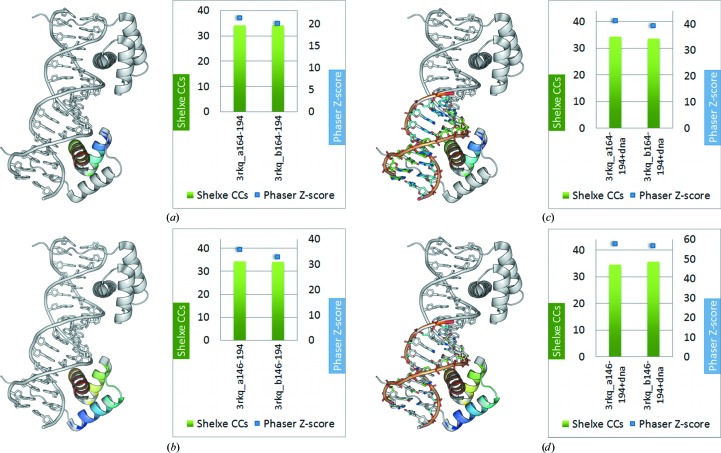
Results for HTH target 3RKQ with ideal fragments: (*a*) HTH fragment (31 residues); (*b*) three-helix bundle fragment (49 residues); (*c*) HTH fragment plus DNA (31 residues + 10 bp); (*d*) three-helix bundle HTH fragment plus DNA (49 residues + 10 bp). With the ideal fragments the target structure 3rkq can easily be solved, as indicated by *SHELXE* CCs of greater than 30% (green bars) and *Phaser* TFZ scores of greater than 20 (blue lines).

For this reason, we decided to perform some tests with ideal fragments for two HTH protein–DNA complexes with available data to a higher resolution (1.7 and 2.0 Å) and containing a smaller fraction of DNA [target structures 3RKQ (Table 2 ▶) and 3PVV (Table 3 ▶)]. For 3RKQ tests were performed on a helix–turn–helix fragment (residues 164–194), a three-helix bundle fragment (residues 146–194) and each of those fragments together with a 10 bp fragment of the double-stranded DNA. Each of the models was provided as a single fragment for an *ARCIMBOLDO* search for two copies. In all of the cases *Phaser* and *SHELXE* are both clearly able to phase and trace the structure correctly (Fig. 10 ▶). Remarkably, the correct location of the ideal models is characterized by notably higher figures of merit than those produced by any of the models in our initial library (LLG of ∼240 *versus* ∼50, TFZ score of ∼20 *versus* 7 for the two-bundle helical fragment and LLG of ∼680 *versus* ∼35, TFZ score of ∼35 *versus* 7 for the three-bundle helical fragment). For 3PVV the ideal fragment chosen was a 8 bp fragment of the DNA and a two-helix bundle fragment of the protein (residues 454–484). Expansion with *SHELXE* resulted in a successful trace, as indicated by a CC of about 30%.

This leads to the conclusion that in the cases of 3RKQ and 3PVV as targets our model library is geometrically too different from the target structures, but that closer models can be recognized by the *Phaser* figures of merit. This suggests that either the models need to be improved, refining internal degrees of freedom against the data, or at least more exhaustive libraries need to be used, either cut out from PDB structures or even varied around these starting points.

#### 3.3.3. HTH new library   

**Figure 11:**
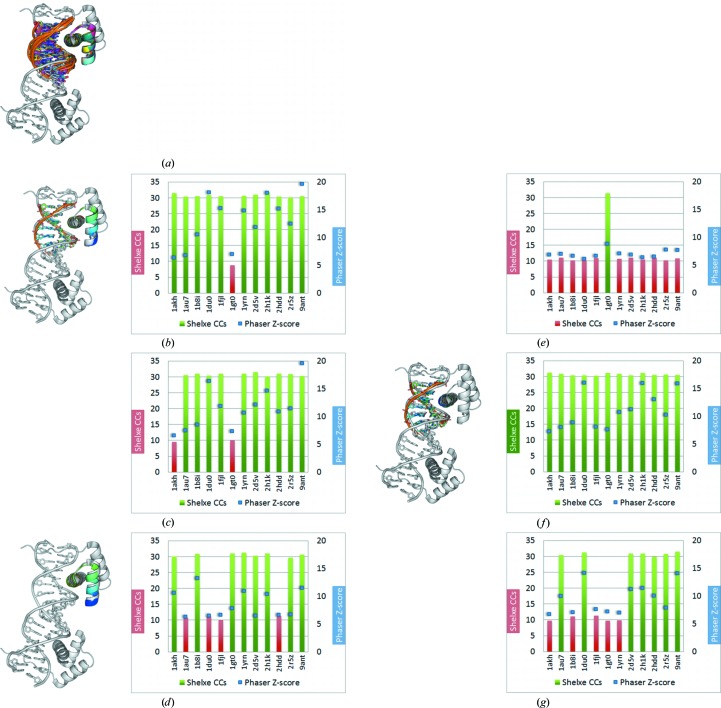
HTH target 3RKQ. On the left side the search models are shown. The right side shows the *Phaser* and *SHELXE* results. Attempts in which *ARCIMBOLDO* succeeds in solving the PDB entry 3RKQ target are shown as green *SHELXE* CC (correlation coefficient) values (fragment PDB codes are listed at the bottom); the *Phaser* TFZ is plotted as blue squares. (*a*) Structure of the target 3RKQ (grey) with all of the models superimposed (coloured). (*b*) HTH fragments without truncation (31–33 amino acids, 7–8 bp); all but one (1gt0) solve the target structure 3RKQ. (*c*) HTH fragments with same number of residues as in (*a*) but with all side chains set to polyalanine; all models except 1akh and 1gt0 solve the target structure. (*d*) HTH fragments without DNA but with the full protein fragment; reducing the phasing information to HTH fragments reduces the number of successful solutions. (*e*) The same HTH fragments as in (*d*) but with polyalanines; one two-helix bundle HTH fragment with polyalanine side chains only solves in the case of 1gt0. (*f*) Models with DNA but only one helix of the protein (the DNA-binding helix); all models can solve the target. (*g*) The same HTH fragments as in (*f*) but polyalanine; without the side chains not all models solve the target structure. The smallest solving fragment represents 3.82% of the mass of the asymmetric unit.

To validate this conclusion, a new library with 12 new subsets of models was generated; their r.m.s.d.s against the 3PVV HTH sites ranged from 3.19 to 0.71 Å and those against 3RKQ were between 0.73 and 0.38 Å. Model subsets comprised the whole HTH motif of 31–33 residues and 7–8 DNA base pairs, the same with side chains truncated to alanine, the protein component of both sets and finally the DNA component bonded to the DNA recognition helix either with or without side chains. Whereas none of these attempts succeeded in solving the 2.0 Å resolution structure, practically all are effective in the case of the more similar, higher resolution 3RKQ (see Tables 2 ▶ and 3 ▶). As can be seen in the results summarized in Fig. 11 ▶, with these more similar sets of fragments either the complete motif (whether truncated to polyalanine or not) or a search fragment constituted by the DNA helix and an α-helix bound to it, succeed in solving the structure in practically all cases, whereas the main chain of the HTH motif devoid of the DNA part is the least effective.

## 4. Conclusions   

Protein–DNA complexes remain a challenging area of macromolecular crystallography. In this work, we explored the suitability of individual DNA-binding protein motifs for solving protein–DNA complex structures using the *ARCIMBOLDO* approach. Zinc-coordinating and zipper-type target structures were solved successfully using protein–DNA specific fragment subsets combined with structure solution *via ARCIMBOLDO* starting from a fragment subset including molecular replacement with *Phaser* and *SHELXE*. However, in the case of the zipper-type complex the long helices already constitute efficient search fragments, an ideal regular helix being close enough to the more tightly wound zipper helix. In this case, a fragment library is clearly unnecessary. On the contrary, in the case of the zinc-finger motif the isolated secondary-structure motifs were not effective while the binding-motifs library was. The method is dependent on sufficiently high-resolution diffraction data, with the limit appearing to be around 2.0 Å. The need for high-resolution data as well as accurate models is highlighted in the third example, where the more variable and challenging helix–turn–helix targets (Fig. 8 ▶) were solved or not depending on these factors. The method is currently limited by *SHELXE* accomplishing expansion from the small fragment to the full structure. However, in favourable cases NCS averaging, as implemented, for example, in the *PHENIX* *AutoBuild* wizard (Terwilliger *et al.*, 2008 ▶), could be used to improve the parameter-to-observation ratio and thereby extend the resolution limits. *Phaser* is generally successful in positioning fragments. Ways to enhance the efficiency of the procedure in the future are suggested by the more accurate models being distinguished by higher figures of merit in *Phaser*, which opens the door to model refinement or library extension. DNA autotracing should also contribute to enhancing the *SHELXE* expansion.

## 5. Related literature   

The following references are cited in the Supporting Information for this article: DeWitt *et al.* (2007 ▶), Elrod-Erickson *et al.* (1998 ▶), Fairall *et al.* (1993 ▶), Fraenkel & Pabo (1998 ▶), Grant *et al.* (2000 ▶), Ha *et al.* (2009 ▶), Houbaviy *et al.* (1996 ▶), Iyaguchi *et al.* (2007 ▶), Jacobson *et al.* (1997 ▶), Joshi *et al.* (2007 ▶), Kim & Berg (1996 ▶), Kumaraswami *et al.* (2009 ▶), LaRonde-LeBlanc *et al.* (2005 ▶), LaRonde-LeBlanc & Wlodawer (2004 ▶), Lee *et al.* (2006 ▶, 2010 ▶), Li *et al.* (1995 ▶, 1998 ▶), Longo *et al.* (2007 ▶), Lu & Klug (2007 ▶), Lu *et al.* (2003 ▶), Miller & Pabo (2001 ▶), Mishra *et al.* (2010 ▶), Nolte *et al.* (1998 ▶), Passner *et al.* (1999 ▶), Pavletich & Pabo (1991 ▶, 1993 ▶), Peisach & Pabo (2003 ▶), Petosa *et al.* (2006 ▶), Poncet-Montange *et al.* (2007 ▶), Reményi *et al.* (2003 ▶), Ren *et al.* (2007 ▶), Schuetz *et al.* (2011 ▶), Segal *et al.* (2006 ▶), Shrivastava & Ramachandran (2007 ▶), Sorenson *et al.* (2004 ▶), Stoll *et al.* (2007 ▶, 2009 ▶), Tahirov *et al.* (2002 ▶), Tucker-Kellogg *et al.* (1997 ▶), Tuske *et al.* (2005 ▶), Wang *et al.* (2001 ▶), Wilson *et al.* (1995 ▶), Wisedchaisri *et al.* (2007 ▶), Wolfe *et al.* (2001 ▶, 2003 ▶), Wu *et al.* (2003 ▶), Yamada *et al.* (2009 ▶) and Zhang *et al.* (2010 ▶, 2011 ▶).

## Supplementary Material

Description of the models used for the three protein-DNA targets zinc-finger, zipper-type and HTH.. DOI: 10.1107/S1399004714007603/rr5060sup1.pdf

## Figures and Tables

**Table 1:** *ARCIMBOLDO* results on zipper-type proteins Several approaches were performed to solve the target structure 2E42 with the fragment models; the TFZ, CC and MPE values in the case of a solution are shown in bold.

	TFZ	CC (%)	MPE (°)
Both helices from the models (30 amino acids)			
1gtw	**20.76**	**28.81**	**50.70**
1h8a	**12.17**	**30.76**	**44.90**
1jnm	6.58	16.02	87.60
2c9l	5.68	16.18	88.80
2h7h	6.24	16.42	87.70
One long helix (30 amino acids) with DNA			
1gtw	**17.36**	**31.66**	**41.30**
1h8a	**8.52**	**29.17**	**49.00**
1jnm	6.76	15.95	88.50
2c9l	5.98	16.22	88.30
2h7h	5.91	15.69	88.60
One long helix (30 amino acids) without DNA			
1gtw	**15.29**	**29.65**	**47.90**
1h8a	**16.71**	**31.35**	**45.00**
1jnm	**9.57**	**24.20**	**54.80**
2c9l	**9.64**	**20.17**	**69.80**
2h7h	**9.56**	**28.27**	**47.30**
Two short helices (12 amino acids) with DNA			
1gtw	**22.97**	**31.86**	**43.50**
1h8a	**13.19**	**30.88**	**51.40**
1jnm	5.88	16.80	88.80
2c9l	5.76	15.70	87.70
2h7h	6.27	16.22	89.00
Only DNA			
1gtw	**9.13**	**28.55**	**48.30**
1h8a	6.52	15.67	89.20
1jnm	6.78	15.64	88.90
2c9l	6.76	16.03	88.90
2h7h	5.96	15.68	89.50
DNA-distant helices			
2e42	**35.21**	**31.71**	**41.70**
Model helix of 30 amino acids			
	**12.68**	**29.22**	**48.50**

**Table 2:** *ARCIMBOLDO* results for HTH proteins Results are shown for several approaches to solve the target structure 3RKQ (115 amino acids and 19 bp) with the fragment models. The TFZ, CC and MPE values in the case of a solution are given in bold. Results are shown after locating two fragments with *Phaser*.

	TFZ	CC (%)	MPE (°)
Full models with DNA and protein with side chains (31–33 amino acids and 7–8 bp)			
1akh	**6.35**	**31.49**	**33.50**
1au7	**6.78**	**30.37**	**33.60**
1b8i	**10.57**	**30.55**	**33.50**
1du0	**18.08**	**30.94**	**33.80**
1fjl	**15.27**	**30.49**	**33.40**
1gt0	7.01	8.83	90.20
1yrn	**14.88**	**30.66**	**33.70**
2d5v	**11.84**	**31.01**	**33.90**
2h1k	**18.07**	**31.93**	**33.50**
2hdd	**15.20**	**30.45**	**33.60**
2r5z	**12.46**	**30.19**	**34.00**
9ant	**19.66**	**30.57**	**33.70**
Full models with DNA and protein without side chains (31–33 amino acids and 7–8 bp)			
1akh	6.59	9.51	89.40
1au7	**7.46**	**30.53**	**33.80**
1b8i	**8.49**	**31.08**	**33.30**
1du0	**16.38**	**30.46**	**33.30**
1fjl	**11.83**	**30.97**	**33.50**
1gt0	7.32	9.96	89.00
1yrn	**10.69**	**31.05**	**33.70**
2d5v	**12.13**	**31.48**	**33.40**
2h1k	**14.63**	**30.23**	**33.40**
2hdd	**10.87**	**31.01**	**33.40**
2r5z	**11.46**	**30.86**	**34.00**
9ant	**19.58**	**30.33**	**33.90**
Models without DNA, protein with side chains (31–33 amino acids)			
1akh	**10.58**	**30.00**	**34.20**
1au7	6.33	10.74	88.90
1b8i	**13.22**	**30.95**	**33.70**
1du0	**6.51**	**11.27**	**73.60**
1fjl	6.62	9.94	89.50
1gt0	**7.83**	**31.06**	**33.40**
1yrn	**10.98**	**31.22**	**33.60**
2d5v	**6.54**	**30.29**	**33.50**
2h1k	**10.37**	**31.04**	**33.90**
2hdd	6.64	11.37	88.50
2r5z	**6.75**	**29.71**	**33.80**
9ant	**11.51**	**30.64**	**34.10**
Models without DNA, protein without side chains (31–33 amino acids)			
1akh	6.86	10.49	89.50
1au7	7.00	11.13	89.40
1b8i	6.62	10.27	89.40
1du0	6.11	10.25	89.20
1fjl	6.68	10.91	89.50
1gt0	**8.79**	**31.34**	**33.30**
1yrn	7.07	10.71	89.70
2d5v	6.84	11.06	89.30
2h1k	6.35	10.44	89.10
2hdd	6.49	11.07	89.50
2r5z	7.74	10.24	89.10
9ant	7.67	10.86	89.30
Models with DNA, only one helix of the protein with side chains (15–17 amino acids and 7–8 bp)			
1akh	**7.29**	**31.33**	**33.30**
1au7	**8.05**	**30.96**	**33.60**
1b8i	**8.85**	**30.45**	**33.90**
1du0	**16.02**	**30.46**	**33.40**
1fjl	**8.10**	**30.27**	**33.30**
1gt0	**7.64**	**31.16**	**33.60**
1yrn	**10.72**	**30.90**	**33.60**
2d5v	**11.25**	**30.38**	**33.90**
2h1k	**15.96**	**31.12**	**33.40**
2hdd	**13.06**	**30.53**	**33.60**
2r5z	**10.29**	**30.71**	**33.50**
9ant	**15.90**	**30.57**	**33.60**
Models with DNA, only one helix of the protein without side chains (15–17 amino acids and 7–8 bp)			
1akh	6.75	9.78	89.30
1au7	**10.01**	**30.44**	**33.30**
1b8i	7.07	11.14	89.10
1du0	**14.17**	**31.28**	**33.80**
1fjl	7.64	11.29	89.20
1gt0	7.18	9.77	89.60
1yrn	6.98	9.82	89.40
2d5v	**11.20**	**30.96**	**33.70**
2h1k	**11.45**	**30.92**	**33.50**
2hdd	**10.02**	**29.89**	**34.10**
2r5z	**7.91**	**30.73**	**33.50**
9ant	**14.09**	**31.54**	**33.10**
Ideal helix (14 amino acids; after location of two fragments)			
	**8.69**	**31.43**	**33.20**

**Table 3:** *ARCIMBOLDO* results for HTH proteins for several approaches to solving the target structure 3PVV with the fragment models The TFZ, CC and MPE values for solutions are given in bold; results are shown after location of two fragments with *Phaser*. Missing fragments did not pass the packing in *Phaser* because of clashes.

	TFZ	CC (%)	MPE (°)
Full models with DNA and protein with side chains			
1akh	9.83	10.11	89.50
1au7	9.65	9.53	89.20
1b8i	9.03	9.49	89.40
1du0	9.67	10.02	89.20
1fjl	9.18	9.47	89.50
1gt0	9.17	8.83	89.20
1yrn	9.93	9.74	89.10
2d5v	8.88	9.16	89.30
2h1k	9.28	9.12	89.40
2hdd	9.18	8.78	89.40
2r5z	9.53	9.23	89.30
9ant	8.76	9.69	89.50
Full models with DNA and protein without side chains			
1akh	10.12	8.90	89.30
1au7	9.68	9.43	89.30
1b8i	10.25	8.54	89.30
1du0	8.74	8.90	89.50
1fjl	9.61	9.12	89.10
1gt0	9.84	8.58	90.00
1yrn	10.28	8.39	89.10
2d5v	9.88	8.57	89.80
2h1k	9.06	9.05	89.30
2hdd	7.90	10.27	89.50
2r5z	9.54	8.89	89.40
9ant	8.56	9.31	89.50
Models without DNA, protein with side chains			
1fjl	8.92		
1yrn	8.23		
2d5v	8.24		
2h1k	10.39		
2hdd	8.13		
2r5z	9.82		
9ant	8.62		
Models without DNA, protein without side chains			
2h1k	11.04		
Models with DNA, only one helix of the protein with side chains			
1akh	11.20	8.61	89.30
1au7	9.73	8.95	89.30
1b8i	9.68	9.61	89.40
1du0	9.26	8.93	89.30
1fjl	10.24	9.56	89.50
1gt0	10.17	9.51	89.20
1yrn	10.86	9.10	89.40
2d5v	10.20	9.19	89.50
2h1k	10.58	8.97	89.50
2hdd	9.57	8.96	89.20
2r5z	10.46	9.70	88.80
9ant	10.68	10.10	89.40
Models with DNA, only one helix of the protein without side chains			
1akh	10.62	9.70	89.30
1au7	10.38	9.49	88.80
1b8i	10.35	9.56	89.50
1du0	10.20	9.56	89.50
1fjl	9.80	9.49	89.70
1gt0	11.50	8.79	89.40
1yrn	10.97	8.54	89.60
2d5v	11.25	9.26	89.40
2h1k	11.07	9.76	89.40
2hdd	8.93	9.32	89.20
2r5z	10.44	8.83	89.60
9ant	10.70	8.87	89.40
Ideal helix (after location of one fragment)			
	11.36		
Perfect fragment (DNA + HTH motif)			
		**27.90**	**41.5**

## Footnotes

1 Supporting information has been deposited in the IUCr electronic archive (Reference: RR5060).

2 PDB fragment 1f2i_h shows a high TFZ score and could be solved successfully using more time-consuming -m300 and -t20 *SHELXE* switches.
