# Supplementary material for: Structure solution of DNA-binding proteins and complexes with ARCIMBOLDO libraries
Source: Acta Crystallogr D Biol Crystallogr. 2014 May 30;70(Pt 6):1743–57. doi: 10.1107/S1399004714007603 (PMC4051508; doi:10.1107/S1399004714007603)
Supplement: Supplementary file 1 [file d-70-01743-sup1.pdf]

Acta Crystallographica Section D

Volume 70 (2014)

Supporting information for article:

Structure solution of DNA binding proteins and complexes with *ARCIMBOLDO* libraries

Kevin Pröpper, Kathrin Meindl, Massimo Sammito, Birger Dittrich, George M. Sheldrick, Ehmke Pohl and Isabel Usón

Table S1 Zinc-finger type.

**PDB**	**Description**	**Res. [Å]**	**R/R_{free} [%]**	**Reference**	**rmsd/No of Cα***	**No of Cα****	**No. in Fig. 3 (Chain)**
1A1G	Zif268-DNA	1.9	0.215/0.27	(Elrod-Erickson *et al.*, 1998)	0.8/30	30	1
1A1H	Zif268-DNA	1.6	0.235/0.277	(Elrod-Erickson *et al.*, 1998)	0.6/30	30	2
1A1I	Zif268-DNA	1.6	0.191/0.216	(Elrod-Erickson *et al.*, 1998)	0.6/29	29	3
1A1J	Zif268-DNA	2.0	0.210/0.262	(Elrod-Erickson *et al.*, 1998)	0.5/30	30	4
1A1K	Zif268-DNA	1.90	0.210/0.259	(Elrod-Erickson *et al.*, 1998)	0.6/30	30	5
1A1L	Zif268-DNA	2.30	0.196/0.267	(Elrod-Erickson *et al.*, 1998)	0.6/30	30	6
1AAY	Zif268-DNA	1.60	0.195/0.242	(Elrod-Erickson *et al.*, 1998)	0.5/30	30	7
1F2I	Zinc-finger-DNA	2.35	0.215/0.256	(Wang *et al.*, 2001)	0.5/31	31	8 (G)<br>9 (H)<br>10 (K)
1G2D	Zinc-finger-DNA	2.20	0.225/0.262	(Wolfe *et al.*, 2001)	0.6/31	31	11 (C)<br>12 (F)
1G2F	Cys2His2 Zinc-finger	2.00	0.233/0.263	(Wolfe *et al.*, 2001)	0.6/31	31	13 (C)
1JK1	Zif-268-DNA	1.90	0.214/0.266	(Miller & Pabo, 2001)	0.5/30	30	14
1JK2	Zif268-DNA	1.65	0.230/0.273	(Miller & Pabo, 2001)	0.7/30	30	15 (A)
1LLM	Zif23-GCN4-DNA	1.5	0.216/0.234	(Wolfe *et al.*, 2003)	0.7/29	29	16 (C)<br>17 (D)
1MEY	Zinc-finger-DNA	2.2	0.224/0.319	(Kim & Berg, 1996)	0.7/29	29	18 (C)<br>19 (F)
1P47	Tandem-Zif-DNA	2.2	0.219/0.267	(Peisach & Pabo, 2003)	0.6/30	30	20 (B)<br>21 (A)

1TF6	TFIIIA-Zif-DNA	3.1	0.308/0.363	(Nolte *et al.*, 1998)	0.9/31	31	22
1UBD	YY1-DNA	2.50	0.212/0.33	(Houbaviy *et al.*, 1996)	0.7/31	31	23
1UN6	Zinc-finger-RNA	3.1	0.216/0.259	(Lu *et al.*, 2003)	1.4/31	30	24 (B)<br>25 (C)<br>26 (D)
1ZAA	Zif268-DNA	2.1	0.182/n.a.	(Pavletich & Pabo, 1991)	0.6/30	30	27
2DRP	Zinc-finger-DNA	2.80	0.193/n.a.	(Fairall *et al.*, 1993)	1.7/29	27	28 (A)<br>29 (D)
2GLI	Five-finger-DNA	2.60	0.228/n.a.	(Pavletich & Pabo, 1993)	0.7/31	31	30
2HGH	TFIII Zinc-finger	NMR	NMR	(Lee *et al.*, 2006)	1.2/31	31	31
2I13	Aart-DNA	1.96	0.203/0.249	(Segal *et al.*, 2006)	0.6/29	29	32 (B)<br>33 (A)
2J7J	Zinc-finger-DNA	1.65	0.218/0.228	(Lu & Klug, 2007)	1.7/31	30	34
2JP9	Wilms-Tumor Zinc-finger-DNA	NMR	NMR	(Stoll *et al.*, 2007)	0.7/29	29	35
2JPA	Wilms Factor Zinc-finger	NMR	NMR	(Stoll *et al.*, 2007)	1.5/31	30	36
2KMK	Gfi-1 Zinc-finger-DNA	NMR	NMR	(Lee *et al.*, 2010)	0.8/29	30	37
2PRT	Wilms-Tumor Zinc-finger-DNA	3.15	0.236/0.277	(Stoll *et al.*, 2007)	0.5/31	31	38
2WBU	KLF4-DNA	2.5	0.220/0.278	(Schuetz *et al.*, 2011)	0.4/31	31	39
3IUF	C2H2 Zinc-finger	1.8	0.195/0.237	(Zhang *et al.*, 2011)	1.6/28	26	40
3MJH	C2H2 Zinc-finger	2.0	0.196/0.260	(Mishra *et al.*, 2010)	1.5/27	27	41 (B)<br>42 (D)

* number of equivalent Cα-atoms used by DALI.

** number of Cα-atoms in the pdb files used for ARCIMBOLDO trials.

Table S2 Zipper-type.

PDB	Description	Res. [Å]	R/R_{free} [%]	reference	No of Cα**
1GTW	C/EPB beta-DNA	1.85	0.231/0.271	PDB	30+29
1H8A	c-Myb/EPB beta-DNA	2.23	0.245/0.288	(Tahirov *et al.*, 2002)	30+30
1JNM	Jun/Cre-DNA	2.20	0.228.0.286	PDB	30+30
2C9L	Zipper-DNA	2.25	0.232/0.264	(Petosa *et al.*, 2006)	30+30
2H7H	Jun BZIP-DNA	2.3	0.224/0.274	PDB	30+30

** number of Cα-atoms in the pdb files used for ARCIMBOLDO trials.

Table S3 Three-helix bundle HTH.

PDB	Description	Res. [Å]	R/R_{free} [%]	reference	rmsd/No of Cα*	No of Cα**
1i6x	Star mutant CRP	2.2	0.230/0.2691	PDB	0.48/14	54
1lj9	SlyA	1.60	0.248/0275	(Wu *et al.*, 2003)	1.05/23	41
1rp3	sigma/anti sigma complex	2.3	0.242/0.262	(Sorenson *et al.*, 2004)	3.21/23	46
1sfx	transcriptional regulator	1.55	0.150/0.183	PDB	3.21/23	41
1tqm	Rio2	1.99	0.174/0.228	(LaRonde-LeBlanc & Wlodawer, 2004)	0.97/26	46
1zar	Rio2 with ADP	1.75	0.185/0.218	(LaRonde-LeBlanc *et al.*, 2005)	0.98/26	46
1zyr	Tt RNA polymerase	3.0	0.261/0.282	(Tuske *et al.*, 2005)		44
2hr3	PA transcriptional regulator	2.4	0.212/0.280	PDB	1.16/26	42
2hyf	BS MntR	2.80	0.217/0.277	(DeWitt *et al.*, 2007)	1.20/26	45
2it0	Ider-DNA	2.60	0.229/0.273	(Wisedchaisri *et al.*, 2007)	1.07/26	45
2ivm	Mtb regulatory protein	2.5	0.201/0.255	(Shrivastava & Ramachandran, 2007)	0.72/26	41
2p6t	NG regulator	2.9	0.239/0.300	(Ren *et al.*, 2007)	1.03/26	40

2zny	FFRP	2.59	0.54/0.258	(Yamada *et al.*, 2009)	0.69/26	41
3cta	Riboflavin kinase	2.20	0.237/0.270	PDB	0.98/26	47
3eco	MepR	2.40	0.242/0.289	(Kumaraswami *et al.*, 2009)	1.17/26	43
3f22	Zalpha-DNA	2.50	0.224/0.269	(Ha *et al.*, 2009)	0.93/26	44
3hrt	ScaR	2.80	0.230/0.282	(Stoll *et al.*, 2009)	1.05/26	45
3jsp	LexA-DNA	2.90	0.267/0.287	(Zhang *et al.*, 2010)	2.65/27	47
3ngo	CNOT6L nuclease	2.20	0.217/0.263§	PDB	0.89/26	43
2q5f	LMNASK1	1.90	0.189/0.222	(Poncet-Montange *et al.*, 2007)	1.1/26	42

* number of equivalent Cα-atoms used by DALI.

** number of Cα-atoms in the pdb files used for ARCIMBOLDO trials.

Table S4 Helix-turn-helix motif plus DNA-fragment

**PDB**	**Description**	**Res [Å]**	**R**	**reference**	**rmsd***
1akh	MATα1/MATα2-DNA	2.50	0.201/0.302	(Li *et al.*, 1998)	0.43
1au7	Pit-1/DNA	2.30	0.230/0.302	(Jacobson *et al.*, 1997)	0.73
1b8i	UBX/EXD-DNA	2.40	0.224/0.304	(Passner *et al.*, 1999)	0.36
1du0	Homeodomain DNA	2.00	0.237/0.270	(Grant *et al.*, 2000)	0.59
1fjl	Drosophila homeodomain-DNA	2.0	0.198/na	(Wilson *et al.*, 1995)	0.39
1gt0	POU/HMG/DNA	2.60	0.232/0.285	(Reményi *et al.*, 2003)	0.53
1yrn	MATα1/MATα2-DNA	2.50	0.225/0.298	(Li *et al.*, 1995)	0.41
2d5v	HNF6α-DNA	2.00	0.247/0.269	(Iyaguchi *et al.*, 2007)	0.63
2h1k	Pdsx homeodomain DNA	2.42	0.227/0.277	(Longo *et al.*, 2007)	0.60
2hdd	Engrailed homeodomain DNA	1.90	0.205/0.251	(Tucker-Kellogg *et al.*, 1997)	0.52
2r5z	Hox-DNA	2.60	0.233/0.303	(Joshi *et al.*, 2007)	0.39
9ant	Antennapeida-homeodomain DNA	2.40	0.218/0.239	(Fraenkel & Pabo, 1998)	0.41

* root-mean-square deviation of 31 equivalent C-alpha atoms treated as one rigid block compared to the correct solution of 3RKQ.

References

DeWitt, M. A., Kliegman, J. I., Helmann, J. D., Brennan, R. G., Farrens, D. L. & Glasfeld, A. (2007). *J. Mol. Biol.* **365**, 1257-1265.
Elrod-Erickson, M., Benson, T. E. & Pabo, C. O. (1998). *Structure* **6**, 451-464.
Fairall, L., Schwabe, J. W. R., Chapman, L., Finch, J. T. & Rhodes, D. (1993). *Nature* **366**, 483-487.
Fraenkel, E. & Pabo, C. O. (1998). *Nat. Struct. Biol.* **5**, 692-697.
Grant, R. A., Rould, M. A., Klemm, J. D. & Pabo, C. O. (2000). *Biochemistry* **39**, 8187-8192.
Ha, S. C., Choi, J., Hwang, H.-Y., Rich, A., Kim, Y.-G. & Kim, K. K. (2009). *Nucleic Acids Res.* **37**, 629-637.
Houbaviy, H. B., Usheva, A., Shenk, T. & Burley, S. K. (1996). *PNAS* **93**, 13577-13582.
Iyaguchi, D., Yao, M., Watanabe, N., Nishihira, J. & Tanaka, I. (2007). *Structure* **15**, 75-83.
Jacobson, E. M., Li, P., Leon-del-Rio, A., Rosenfeld, M. G. & Aggarwal, A. K. (1997). *Genes Dev.* **11**, 198-212.
Joshi, R., Passner, J. M., Rohs, R., Jain, R., Sosinsky, A., Crickmore, M. A., Jacob, V., Aggarwal, A. K., Honig, B. & Mann, R. S. (2007). *Cell* **131**, 530-543.
Kim, C. A. & Berg, J. M. (1996). *Nat. Struct. Biol.* **3**, 940-945.
Kumaraswami, M., Schuman, J. T., Seo, S. M., Kaatz, G. W. & Brennan, R. G. (2009). *Nucleic Acids Res.* **37**, 1211-1224.
LaRonde-LeBlanc, N., Guszczynski, T., Copeland, T. & Wlodawer, A. (2005). *FEBS Journal* **272**, 2800-2810.
LaRonde-LeBlanc, N. & Wlodawer, A. (2004). *Structure* **12**, 1585-1594.
Lee, B. M., Xu, J., Clarkson, B. K., Martinez-Yamout, M. A., Dyson, H. J., Case, D. A., Gottesfeld, J. M. & Wright, P. E. (2006). *J. Mol. Biol.* **357**, 275-291.
Lee, S., Doddapaneni, K., Hogue, A., McGhee, L., Meyers, S. & Wu, Z. (2010). *J. Mol. Biol.* **397**, 1055-1066.
Li, T., Jin, Y., Vershon, A. K. & Wolberger, C. (1998). *Nucleic Acids Res.* **26**, 5707-5718.
Li, T., Stark, M. R., Johnson, A. D. & Wolberger, C. (1995). *Science* **270**, 262-269.
Longo, A., Guanga, G. P. & Rose, R. B. (2007). *Biochemistry* **46**, 2948-2957.
Lu, D. & Klug, A. (2007). *Proteins* **67**, 508-512.
Lu, D., Searles, M. A. & Klug, A. (2003). *Nature* **426**, 96-100.
Miller, J. C. & Pabo, C. O. (2001). *J. Mol. Biol.* **313**, 309-315.
Mishra, A., Eathiraj, S., Corvera, S. & Lambright, D. G. (2010). *PNAS* **107**, 10866-10871.
Nolte, R. T., Conlin, R. M., Harrison, S. C. & Brown, R. S. (1998). *PNAS* **95**, 2938-2943.
Passner, J. M., Ryoo, H. D., Shen, L., Mann, R. S. & Aggarwal, A. K. (1999). *Nature* **397**, 714-719.
Pavletich, N. P. & Pabo, C. O. (1991). *Science* **252**, 809-817.
Pavletich, N. P. & Pabo, C. O. (1993). *Science* **261**, 1701-1707.
Peisach, E. & Pabo, C. O. (2003). *J. Mol. Biol.* **330**, 1-7.
Petosa, C., Morand, P., Baudin, F., Moulin, M., Artero, J.-B. & Müller, C. W. (2006). *Mol. Cell* **21**, 565-572.
Poncet-Montange, G., Assairi, L., Arold, S., Pochet, S. & Labesse, G. (2007). *J. Biol. Chem.* **282**, 33925-33934.
Reményi, A., Lins, K., Nissen, L. J., Reinbold, R., Schöler, H. R. & Wilmanns, M. (2003). *Genes Dev.* **17**, 2048-2059.
Ren, J., Sainsbury, S., Combs, S. E., Capper, R. G., Jordan, P. W., Berrow, N. S., Stammers, D. K., Saunders, N. J. & Owens, R. J. (2007). *J. Biol. Chem.* **282**, 14655-14664.
Schuetz, A., Nana, D., Rose, C., Zocher, G., Milanovic, M., Koenigsmann, J., Blasig, R., Heinemann, U. & Carstanjen, D. (2011). *Cell. Mol. Life Sci.* **68**, 3121-3131.
Segal, D. J., Crotty, J. W., Bhakta, M. S., Barbas, C. F., III & Horton, N. C. (2006). *J. Mol. Biol.* **363**, 405-421.
Shrivastava, T. & Ramachandran, R. (2007). *Nucleic Acids Res.* **35**, 7324-7335.
Sorenson, M. K., Ray, S. S. & Darst, S. A. (2004). *Mol. Cell* **14**, 127-138.

Stoll, K. E., Draper, W. E., Kliegman, J. I., Golynskiy, M. V., Brew-Appiah, R. A. T., Phillips, R. K., Brown, H. K., Breyer, W. A., Jakubovics, N. S., Jenkinson, H. F., Brennan, R. G., Cohen, S. M. & Glasfeld, A. (2009). *Biochemistry* **48**, 10308-10320.
Stoll, R., Lee, B. M., Debler, E. W., Laity, J. H., Wilson, I. A., Dyson, H. J. & Wright, P. E. (2007). *J. Mol. Biol.* **372**, 1227-1245.
Tahirov, T. H., Sato, K., Ichikawa-Iwata, E., Sasaki, M., Inoue-Bungo, T., Shiina, M., Kimura, K., Takata, S., Fujikawa, A., Morii, H., Kumasaka, T., Yamamoto, M., Ishii, S. & Ogata, K. (2002). *Cell* **108**, 57-70.
Tucker-Kellogg, L., Rould, M. A., Chambers, K. A., Ades, S. E., Sauer, R. T. & Pabo, C. O. (1997). *Structure* **5**, 1047-1054.
Tuske, S., Sarafianos, S. G., Wang, X., Hudson, B., Sineva, E., Mukhopadhyay, J., Birktoft, J. J., Leroy, O., Ismail, S., Clark, A. D., Jr., Dharia, C., Napoli, A., Laptenko, O., Lee, J., Borukhov, S., Ebright, R. H. & Arnold, E. (2005). *Cell* **122**, 541-552.
Wang, B. S., Grant, R. A. & Pabo, C. O. (2001). *Nat. Struct. Biol.* **8**, 589-593.
Wilson, D. S., Guenther, B., Desplan, C. & Kuriyan, J. (1995). *Cell* **82**, 709-719.
Wisedchaisri, G., Chou, C. J., Wu, M., Roach, C., Rice, A. E., Holmes, R. K., Beeson, C. & Hol, W. G. J. (2007). *Biochemistry* **46**, 436-447.
Wolfe, S. A., Grant, R. A., Elrod-Erickson, M. & Pabo, C. O. (2001). *Structure* **9**, 717-723.
Wolfe, S. A., Grant, R. A. & Pabo, C. O. (2003). *Biochemistry* **42**, 13401-13409.
Wu, R.-Y., Zhang, R.-G., Zagnitko, O., Dementieva, I., Maltzev, N., Watson, J. D., Laskowski, R., Gornicki, P. & Joachimiak, A. (2003). *J. Biol. Chem.* **278**, 20240-20244.
Yamada, M., Ishijima, S. A. & Suzuki, M. (2009). *Proteins* **74**, 520-525.
Zhang, A. P. P., Pigli, Y. Z. & Rice, P. A. (2010). *Nature* **466**, 883-886.
Zhang, W., Xu, C., Bian, C., Tempel, W., Crombet, L., MacKenzie, F., Min, J., Liu, Z. & Qi, C. (2011). *Biochem. Biophys. Res. Commun.* **413**, 58-61.
